# Effect of Implant Angulation on the Rotational Displacement of a 3-Unit Bridge after Digital Impression

**DOI:** 10.1155/2022/8634091

**Published:** 2022-01-25

**Authors:** Mahnaz Arshad, Amirmohsen Asgari, Mohamad Javad Kharazifard, Narges Ameri

**Affiliations:** ^1^Dental Research Center, Dentistry Research Institute, Tehran University of Medical Sciences, Tehran, Po. Code: 14399-55991, Iran; ^2^Department of Prosthodontics, School of Dentistry, International Campus, Tehran University of Medical Sciences, Tehran, Po. Code:14399-55991, Iran; ^3^Private Practice, Tehran, Iran; ^4^Department of Epidemiology and Biostatistics, Faculty of Public Health, Dental School, Tehran University of Medical Sciences, Tehran, Po. Code:14399-55991, Iran

## Abstract

**Objectives:**

This study aimed to assess the effect of implant angulation on the rotational displacement of a 3-unit bridge following a digital impression.

**Materials and Methods:**

This in vitro experimental study evaluated 3 master models of the maxilla with Kennedy's class III partial edentulism and bilateral three-unit implant-supported fixed partial dentures. Two implants were placed with 0° (first model), 15° (second model), and 30° (third model) interimplant angles. The implants were placed bilaterally at the sites of first premolars and first molars from the posterior towards the anterior region and coded A (posterior) and B (anterior) in the left, and C (posterior) and D (anterior) in the right side. Next, their position was recorded using a coordinate measuring machine to serve as a reference. The models were then scanned by both 3Shape and Sirona digital scanners (12 times by each scanner). The obtained data were compared with the reference data three-dimensionally using GOM Inspect software to determine the rotational displacement of the implants. Data were analyzed by repeated-measures ANOVA, one-way ANOVA, paired sample *t*-test, and independent sample *t*-test (*P* < 0.05).

**Results:**

Since repeated-measures ANOVA showed that the interaction effect was significant (*P* = 0.010), the data were analyzed by subgroup analysis. The 3Shape scanner showed significantly higher accuracy for C-D region in model 2 (*P* = 0.001), and A-B region in model 1 (*P* ≤ 0.01). In the use of the 3Shape scanner, model 3 showed a lower error rate in the A-B region, compared with models 1 and 2. Model 1 showed higher error rate than models 2 and 3 in the C-D region (*P* ≤ 0.01). In the use of the Sirona scanner, model 1 showed a higher error rate than models 2 (*P* = 0.031) and 3 (*P* = 0.004) in the C-D region.

**Conclusion:**

In digital impressions of angulated implants in 3-unit bridges by using 3Shape and Sirona scanners, the rotational error decreases as the interimplant angle increases.

## 1. Introduction

Implant-restoration misfits can cause biological and mechanical complications such as marginal bone loss, gingival inflammation, screw loosening, and implant failure. Thus, passive fit is imperative for the long-term success of restorations [1]. Recording the interimplant relationship by a reliable impression technique is the first step in achieving a prosthetic restoration with passive fit [2]. In implant impressions, recording the three-dimensional (3D) position of implants relative to each other is more important than recording the superficial details to ensure treatment success [3]. The advances in intraoral optical impression systems and the advent of computer-aided design/computer-aided manufacturing (CAD/CAM) techniques increased digitization in dental clinical procedures, such that at present, intraoral scanners and the CAD/CAM technology are extensively used as an alternative to routine conventional procedures [4, 5].

All-digital CAD/CAM systems include an intraoral scanner, a computer with CAD software, and a milling machine [6]. Intraoral scanners have greatly evolved since their introduction to the dental market [7]. The currently available intraoral scanners include CEREC Omnicam (Dentsply Sirona), iTero Element (Align Technology), Planmeca PlanScan (E4D Technologies), Trios (3shape), and 3M True Definition (3M ESPE) [8]. It appears that implant-supported restorations fabricated by the CAD/CAM technology have a survival rate comparable to that of conventionally fabricated restorations [9].

The literature is controversial regarding the accuracy of intraoral scanners; some authors [10–12] reported an accuracy similar to or higher than that of conventional impression materials such as polyvinyl siloxane, while some others [13, 14] reported lower accuracy of intraoral scanners compared with that of conventional impressions.

Fully digital fabrication of prosthetic dental restorations has several advantages such as improved patient cooperation, standardized accuracy, real-time imaging and demonstration, enhanced patient-physician communication, and improved time efficiency compared with the conventional technique [9, 15–18].

Digital implant impressions are made by using scan bodies and intraoral scanners, which eliminate the need for a tray and impression material, as well as the need for tightening and opening of impression copings. Also, this technique does not require a wide mouth opening, which is required for impression making with impression copings [19]. Digital implant impressions require a scan body attached to the implant as well as an intraoral scanner to record the surface topography of the scan body and oral structures. The geometric property of the scan body provides some information about the implant orientation, angulation, and 3D position. Next, the scanned data are processed by the scanner software, and the next steps are followed using the digital database of the respective implant [20].

One factor for assessment of the 3D position is the rotational angle of the scan body around the implant axis, which is important in the fabrication of multiunit bridges to achieve passive fit. This angle can be measured according to the prepared (reduced) oblique surface (s) of the scan body [21].

Several potential factors can affect the accuracy of conventional implant impressions including the impression material, the connection of different implant components, and the machining tolerance. In different clinical scenarios, depending on the condition, parallel placement of implants may be difficult due to anatomical limitations. In such cases, the implant angulation may range from 5° to 40° [22]. In the clinical setting, the degree of divergence or convergence of implants is often >8°–10°. When multiple implants with different angulations are placed, deformation of the impression material may increase upon tray removal. It has been reported that angulated implants yield poorer results in comparison with parallel implants in the assessment of dental casts with 4 or 5 implants [23, 24]. Also, evidence shows that implant angulation can increase inaccuracy if the interimplant angle is ≥ 15° [25]. Angulated implants pose a clinical challenge due to anatomical limitations, esthetic considerations, and operator-related factors. Digital impressions can eliminate some of the problems related to angulated implants and may bring about higher accuracy [25].

Nonetheless, evidence shows that digital scanners are comparable or even superior to conventional impressions. However, the results on this topic are controversial. Also, there is a gap of knowledge about the accuracy of digital scanners for dental implants, especially for the fabrication of bridges [26]. Information to support the CAD/CAM technology for dental rehabilitation with implants is lacking, and high-quality studies are required to confirm its efficacy [27]. Considering the gap of information regarding the effect of implant angulation on the accuracy of intraoral scanners for 3-unit bridges, this study aimed to assess the effect of implant angulation on the accuracy of digital impressions in terms of rotational displacement in 3-unit bridges.

## 2. Materials and Methods

In this in vitro experimental study, the sample size was calculated to be 12 according to a study by Giménez et al. [28] using the one-way ANOVA Power Analysis feature of PASS 11 software, assuming alpha = 0.05, beta = 0.2, standard deviation of 24.80, and an effect size of 0.51. A sample size of 12 in this study indicated 12 repetitions of scanning of each cast by each scanner.

### 2.1. Fabrication of Master Model

Three master models of the maxilla with Kennedy's class III (modification I) bilateral partial edentulism at the sites of first premolar to first molar were fabricated from resin and used in this study.

### 2.2. Implant Placement

The angulation of implant fixtures was determined using a caliper. For implant fixture placement, the implant holes were created in the model with a bur. The edentulous area was measured to be 24 mm using a digital caliper (Extra Strong, China). The center of the created implant hole was 4 mm away from the adjacent tooth. In each model, four implant fixtures (BLT ITI, Straumann, Switzerland) with a 4.1 diameter and a 10 mm length were bilaterally placed at the sites of the first premolars and first molars. A total of 12 implants with 0°, 15°, and 30° interimplant angles were placed. In all three models, the implants were placed from the posterior towards the anterior region and coded A (posterior) and B (anterior) in the left, and C (posterior) and D (anterior) in the right side.

In the first model, in each quadrant, the implants were mounted parallel to each other at a 0° angle relative to each other.

In the second model, in the left side, implant A had a 0° angulation and implant B had a 15° angulation, with a total interimplant angle of 15° relative to each other. In other words, the implants were divergent in the left side. In the right side, implant C had a 15° angle and implant B had a 0° angle with a total interimplant angle of 15° relative to each other. In other words, the implants were divergent in the right side.

**Figure 1:**
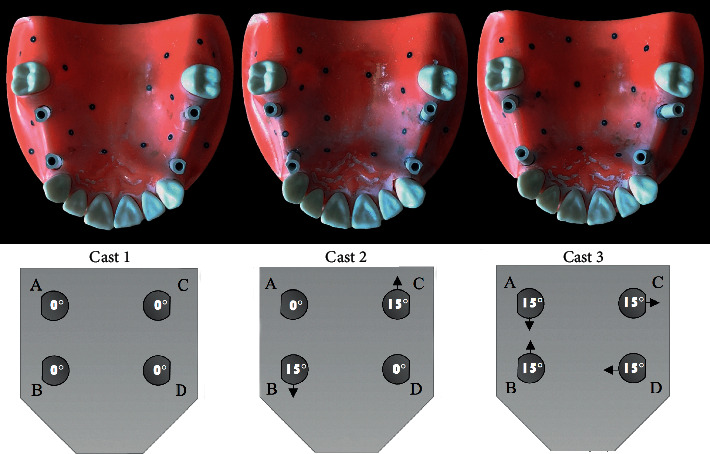
Implant angulations in the three models.

In the third model, all implants were mounted at a 15° angle with a total interimplant angle of 30° on each side. In the left side, the implants were placed mesiodistally and were convergent while in the right side, they were placed buccolingually and were divergent (Figure 1).

### 2.3. Scanning of the Models

A scan body (Straumann) was tightened to each implant. The STL file of the master reference model was obtained by an industrial light scanner (ATOS Core 80 5MP; GOM GmbH, Braunschweig, Germany), and then digitized by metrology software (Pro 8.1; GOM GmbH, Braunschweig, Germany). The sphere space error of the scanner was 6 *μ*m, and its size error was 8 *μ*m [29]. A thin layer (2 *μ*m) of antireflective powder was sprayed on the surface of the model before scanning [30]. This scan served as the reference scan for the purpose of comparison. The data were saved in g3d format for measurements and later compared using the GOM Inspect software [31, 32].

Each master model with scan bodies tightened to the implants was first scanned by the TRIOS intraoral scanner (3Shape, Denmark) 12 times and then with the CEREC Omnicam intraoral scanner (Sirona Dental, USA) 12 times. An experienced operator performed all the scanning procedures. Data were saved in STL format. Eventually, 72 STL files of the GOM Inspect software were three dimensionally evaluated and compared with the files obtained from the ATOS Triple Scan to compare the implant angulations.

**Figure 2:**
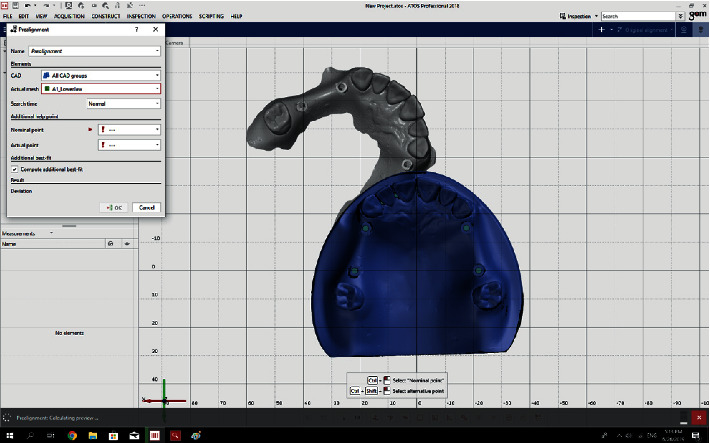
The prealignment feature was selected from the drop-down menu in the GOM Inspect software.

**Figure 3:**
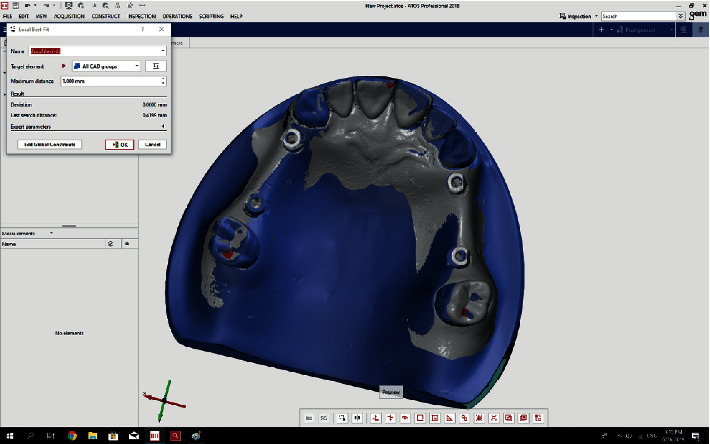
The local best-fit feature was selected from the menu in GOM Inspect.

**Figure 4:**
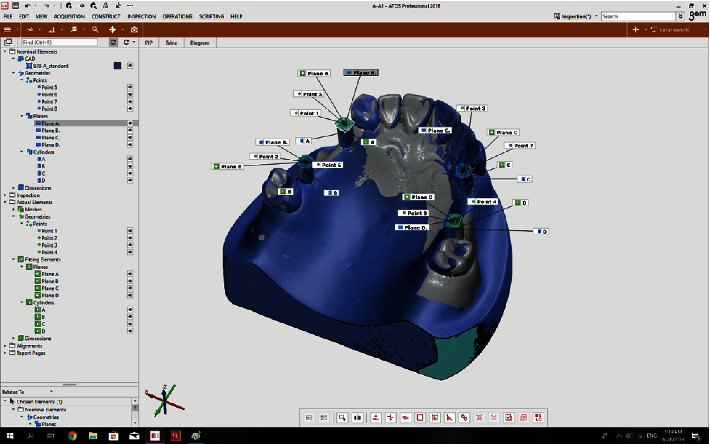
Assessment and comparison of the scanned models with the reference scan.

**Figure 5:**
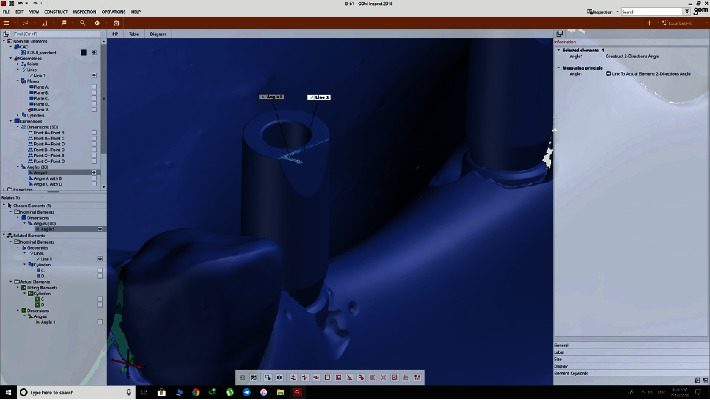
Determination of the angle of rotation around the longitudinal axis of the scan body.

The GOM Inspect software (ATOS) version 2018 compared each of the 72 STL files (36 scans by 3Shape and 36 scans by Sirona) with the reference files as follows:

- The two files to be compared were selected.
- The prealignment feature was selected from the drop-down menu to primarily record the 3D coordinates of the models (Figure 2).
- The local best-fit feature was then selected to choose three regions for final superimposition of the models (Figure 3).
- A superimposition was performed for each of the 72 models (Figure 4).
- The difference in angle of rotation around the longitudinal axis of the scan body was determined as follows: a line formed at the intersection of the upper surface of the scan body and its tapered surface (that indicated the direction of the scan body) was used as an index for implant rotation around its longitudinal axis. The difference between the angulation of this line with the same line drawn for the reference scan body was calculated and reported (Figure 5).

The measurements were repeated twice by the same operator with a one-week interval, and the intraclass correlation coefficients were calculated to be 0.94 and 0.97 for 3Shape and Sirona, respectively. Data were collected in Excel software version 16 and analyzed using SPSS version 25 (SPSS Inc., IL, USA). The effects of cast type, scanner type, and region were evaluated using repeated-measures ANOVA considering cast type and region as between-subject factors. All of the interaction effects were significant (*P* < 0.05). Therefore, subgroup analysis was applied. One-way ANOVA was used in order to compare the cast types. The independent sample *t*-test was used to compare the regions, and the paired sample *t*-test was applied to compare Sirona and 3Shape scanners. The level of significance was set at 0.05.

## 3. Results

Table 1 shows the mean and standard deviation of rotational displacement of implants in the three models separately for the two scanners. Since repeated-measures ANOVA showed that the interaction effect was significant (*P* = 0.010), the data were analyzed by subgroup analysis.

Table 2 compares the two scanners separately for each model in the right (A-B) and left (C-D) quadrants. According to the paired sample *t*-test, the C-D region in the second model (*P* = 0.001) and the A-B region in the third model (*P* ≤ 0.01) showed significant differences between the two scanners, and 3Shape demonstrated higher accuracy in these areas.

Comparison of the accuracy of the right (A-B) and left (C-D) side implants in each model between the two scanners by the independent sample *t*-test revealed a significant difference in the accuracy of scanning of the right and left quadrants by 3Shape in the second model (*P* ≤ 0.01) and by Sirona in the third model (*P* = 0.001), such that the error rate was higher in the A-B region. No other significant differences were noted (*P* > 0.05).

Table 3 compares the accuracy of the three casts with each other using one-way ANOVA. As shown, in the use of the 3Shape scanner, models 1 (*P* ≤ 0.01) and 2 (*P* ≤ 0.01) had significantly higher error rates than model 3 in the A-B region. Model 1 had a significantly higher error rate than models 2 (*P* ≤ 0.01) and 3 (*P* ≤ 0.01) in the C-D region.

In the use of the Sirona scanner, no significant difference was noted between the models in the A-B region. Model 1 had a significantly higher error rate than models 2 (*P* = 0.031) and 3 (*P* = 0.004) in the C-D region.

## 4. Discussion

For accurate treatment planning, knowledge about the effect of implant angulation on the accuracy of digital impression technique is imperative because straight implant placement is not feasible in many clinical scenarios [33]. On the other hand, an accurate and reliable impression is required to achieve optimal fit in implant-supported restorations [34]. In general, errors that occur in the process of impression making such as linear errors, angular errors, or rotational displacement result in incomplete seating of the bridge over the implants, and may impair the passive fit and lead to biomechanical and biological complications in dental implants and peri-implant tissues. Therefore, such errors should be minimized [35–38]. Controversy exists regarding the level of acceptable inaccuracy for implant-supported restorations. In general, implant-supported restorations require higher precision than tooth-supported restorations. Thus, the clinician and technician should make a greater effort for the fabrication of a highly accurate prosthetic restoration. Making a precise impression is of great importance in this process [39, 40]. However, some levels of error are acceptable and do not cause clinical, prosthetic, or biological complications, which is referred to as biological tolerance [41]. According to the literature, the acceptable linear threshold is 100 µm, and the acceptable angle of error is 0.2–0.5 degrees [40, 42, 43].

This study assessed the effect of implant angulation in a three-unit bridge on rotational displacement following digital impression making by the use of 3Shape Trios and Sirona intraoral scanners. The accuracy of an impression is determined based on its precision and trueness. Precision refers to the similarity of repeated measurements. Higher accuracy brings about more predictable measurements. Trueness refers to the level of similarity of measurements to the actual dimensions of the respective object. A high level of trueness indicates high similarity to the actual dimensions of the respective object [44]. This study evaluated the precision and trueness of digital impression making by intraoral scanners by assessing the rotational displacement of angulated implants in a 3-unit bridge.

The impression material, technique of impression, operator errors, and incorrect connection of implant components can affect the success of implant-supported restorations. These problems may be minimized by digital impression making [45]. This study assessed the deviation of data obtained by 3Shape and Sirona intraoral scanners from the actual model. In the use of the 3Shape scanner, in the A-B region, the minimum error was noted in model 3 (30° angle). In the C-D region, the maximum error was noted in model 1 (0° angle). In the use of the Sirona scanner in the A-B region, implant angulation had no significant effect on impression accuracy. However, in the C-D region, the maximum error was noted in model 1 (0° angle). According to the results, it appears that by an increase in interimplant angle, the impression accuracy either increased in terms of rotational displacement or the interimplant angle had no significant effect on the impression accuracy (in the A-B region in the use of the Sirona scanner).

Lin et al. [46] evaluated the accuracy of digital impressions of implants with 0, 15, 30, and 45° divergence angles and concluded that as the interimplant angle increased, deviation in distance and angulation decreased. Also, Ribeiro et al. [47] concluded that increasing the interimplant angle did not increase the distance deviation, and even in some cases with angulated implants, digital impressions showed a higher accuracy. The findings of Lin et al. [46] and Ribeiro et al. [47] were in line with the present results. However, Giménez et al. [28] evaluated the accuracy of full-arch digital impressions made by the Lava oral scanner for dental implants with 30-degree mesial and distal angulations. They observed that implant angulation had no significant effect on the accuracy of digital impressions. Chia et al. [25] noticed that with an increase in buccolingual angulation (0, 10, and 20 degrees), the accuracy of digital impressions decreased. Also, Arcuri et al. [48] assessed the effect of 0–20-degree implant angulations on the accuracy of full-arch digital impressions made by the 3Shape scanner. They found that the interimplant angle significantly affected the linear deviation.

A comparison of 3Shape and Sirona scanners regarding the effect of interimplant angle on rotational displacement of implants revealed that the 3Shape scanner had a significantly lower error rate than the Sirona at an interimplant angle of 0° at the site of starting the scan and 15° when the posterior implant was angulated. Also, in the 30° mesiodistally convergent position of implants, the 3Shape scanner showed a significantly lower error rate than the Sirona scanner. However, no significant difference was noted between the two scanners in other positions. Malik et al. [49] found no significant difference in the accuracy of 3Shape and Sirona scanners. Ferrini et al. [50] compared the accuracy of intraoral and extraoral scanners for single-unit abutments and found that both scanners had acceptable clinical performance regarding marginal fit, but the Sirona scanner had higher accuracy than 3Shape.

In models 2 and 3 of the present study, the site of initiation of scanning (C-D region) showed higher accuracy than the site of termination of scanning, which was in agreement with the results of Kim et al. [51]. Also, Giménez et al. [28] compared the accuracy of Lava COS and iTero scanners and observed that the error rate increased as moving from the site of initiation of scanning of implants towards the posterior region of a model of the maxilla with 6 implants, which was in agreement with the present findings.

In this study, comparison of the accuracy of implants between the right and left quadrants of the first model (bilateral implants with 0° interimplant angle) revealed no significant difference in the use of 3Shape or Sirona intraoral scanners. In the second model in our study, the 3Shape scanner showed higher accuracy when the interimplant angle was at 15°, and the posterior implant was angulated. However, no significant difference was noted in this respect in the use of Sirona. In the third model in our study, the Sirona scanner showed higher accuracy for implants placed buccolingually with an interimplant angle of 30° compared with mesiodistally placed implants. However, in the use of the 3shape scanner, no difference was noted in the accuracy of buccolingually and mesiodistally placed implants.

However, it should be noted that since none of the abovementioned studies evaluated rotational displacement, their results cannot be accurately compared with the present findings. Also, a recent review study by Sanda et al. [40] concluded that a definite conclusion cannot be drawn regarding the effect of implant angulation on the accuracy of digital impressions since the available studies on this topic have used different types of scanners, have evaluated different types of errors, and have used different instruments for assessment of accuracy.

This study had some limitations. It only assessed the accuracy of two intraoral scanners. The accuracy of digital impressions by other intraoral scanners should also be evaluated in future studies. Other implant angulations should be studied as well. Considering the in vitro design of this study, generalization of results to the clinical setting must be done with caution. Also, future studies should assess the effect of errors from the initiation to the end of scanning by intraoral scanners on rotational displacement of angulated implants. Clinical trials are required to assess the role of implant angulation and different implant systems and compare the accuracy of different impression techniques with different impression materials. Last but not least, the accuracy of digital impressions of other forms of complete and partial edentulism, and single-unit, multiunit, and bridge restorations should be investigated.

## 5. Conclusion

Within the limitations of this study, the results indicated an increase in rotational displacement in most areas of digital impressions by an increase in interimplant angle. The error rate at the site of initiation of scanning was lower than that at the site of termination of scanning. Also, the two scanners had significantly different digital impression accuracy for angulated implants, and the accuracy of Sirona was comparable and sometimes lower than that of 3Shape.

## Figures and Tables

**Table 1:** The mean and standard deviation of rotational displacement (in degrees) of implants in the three models scanned by the two scanners (*n* = 12).

Cast	Region	Scanner	Minimum	Maximum	Mean	Std. deviation
1	A-B	3Shape	0.80	1.20	0.9833	0.14668
		Sirona	0.50	1.60	1.0167	0.35119
	C-D	3Shape	0.40	1.20	0.8000	0.26968
		Sirona	0.70	1.50	1.0083	0.24293

2	A-B	3Shape	0.40	1.10	0.8250	0.18647
		Sirona	0.40	1.50	0.8083	0.34234
	C-D	3Shape	0.10	0.50	0.3083	0.13114
		Sirona	0.40	1.30	0.7083	0.24664

3	A-B	3Shape	0.10	0.60	0.4250	0.18153
		Sirona	0.70	1.50	1.0500	0.24680
	C-D	3Shape	0.10	0.80	0.3750	0.20505
		Sirona	0.10	1.00	0.6167	0.32983

**Table 2:** Comparison of the two scanners separately in each model and in the right and left quadrants using the paired sample *t*-test.

Model	Region		Scanners	*P* value
1	A-B	Pair	3Shape and Sirona	0.692
	C-D	Pair	3Shape and Sirona	0.096

2	A-B	Pair	3Shape and Sirona	0.881
	C-D	Pair	3Shape and Sirona	0.001

3	A-B	Pair	3Shape and Sirona	≤0.01
	C-D	Pair	3Shape and Sirona	0.071

**Table 3:** Comparison of the accuracy of the two scanners in the three models bilaterally using one-way ANOVA.

Region	Scanner	Model (I)	Model (J)	*P* value
A-B	3Shape	1	2	0.078
			3	≤0.01
		2	1	0.078
			3	≤0.01
		3	1	≤0.01
			2	≤0.01
	Sirona	1	2	0.256
			3	0.964
		2	1	0.256
			3	0.164
		3	1	0.964
			2	0.146

C-D	3Shape	1	2	≤0.01
			3	≤0.01
		2	1	≤0.01
			3	0.719
		3	1	≤0.01
			2	0.719
	Sirona	1	2	0.031
			3	0.698
		2	1	0.004
			3	0.698
		3	1	0.004
			2	0.698

## Data Availability

The data used to support the findings of this study were supplied by Narges Ameri under license and thus cannot be made freely available. Requests for access to these data should be made to Narges Ameri.
